# Cost Effectiveness of Potential ART Adherence Monitoring Interventions in Sub-Saharan Africa

**DOI:** 10.1371/journal.pone.0167654

**Published:** 2016-12-15

**Authors:** Andrew N Phillips, Valentina Cambiano, Fumiyo Nakagawa, Loveleen Bansi-Matharu, Papa Salif Sow, Peter Ehrenkranz, Deborah Ford, Owen Mugurungi, Tsitsi Apollo, Joseph Murungu, David R. Bangsberg, Paul Revill

**Affiliations:** 1 Research Department of Infection & Population Health, UCL, London, United Kingdom; 2 Bill & Melinda Gates Foundation, Seattle, Washington, United States of America; 3 Institute for Clinical Trials and Methodology, UCL, London, United Kingdom; 4 Ministry of Health & Child Care, Harare, Zimbabwe; 5 Oregon Health Sciences University-Portland State University School of Public Health, Portland, Oregon, United States of America; 6 Centre for Health Economics, University of York, York, United Kingdom; International AIDS Vaccine Initiative, UNITED STATES

## Abstract

**Background:**

Interventions based around objective measurement of adherence to antiretroviral drugs for HIV have potential to improve adherence and to enable differentiation of care such that clinical visits are reduced in those with high adherence. It would be useful to understand the approximate upper limit of cost that could be considered for such interventions of a given effectiveness in order to be cost effective. Such information can guide whether to implement an intervention in the light of a trial showing a certain effectiveness and cost.

**Methods:**

An individual-based model, calibrated to Zimbabwe, which incorporates effects of adherence and resistance to antiretroviral therapy, was used to model the potential impact of adherence monitoring-based interventions on viral suppression, death rates, disability adjusted life years and costs. Potential component effects of the intervention were: enhanced average adherence when on ART, reduced risk of ART discontinuation, and reduced risk of resistance acquisition. We considered a situation in which viral load monitoring is not available and one in which it is. In the former case, it was assumed that care would be differentiated based on the adherence level, with fewer clinic visits in those demonstrated to have high adherence. In the latter case, care was assumed to be primarily differentiated according to viral load level. The maximum intervention cost required to be cost effective was calculated based on a cost effectiveness threshold of $500 per DALY averted.

**Findings:**

In the absence of viral load monitoring, an adherence monitoring-based intervention which results in a durable 6% increase in the proportion of ART experienced people with viral load < 1000 cps/mL was cost effective if it cost up to $50 per person-year on ART, mainly driven by the cost savings of differentiation of care. In the presence of viral load monitoring availability, an intervention with a similar effect on viral load suppression was cost-effective when costing $23-$32 per year, depending on whether the adherence intervention is used to reduce the level of need for viral load measurement.

**Conclusion:**

The cost thresholds identified suggest that there is clear scope for adherence monitoring-based interventions to provide net population health gain, with potential cost-effective use in situations where viral load monitoring is or is not available. Our results guide the implementation of future adherence monitoring interventions found in randomized trials to have health benefit.

## Introduction

Various potential means to improve adherence to antiretroviral therapy have been evaluated in sub-Saharan Africa [1,2]. Most involve a component of counselling and/or support by clinic staff or community based counsellors, which often takes place face-to-face but can also include phone calls or text messaging. A key challenge with any such intervention is having access to a reliable and objective indicator of the extent and pattern of the patient’s adherence. Objective measures of adherence that do not rely on self-report are attractive in not being subject to reporting bias and as such they have potential for routine use as a means of informing and targeting interventions to improve adherence. Objective adherence measurement approaches include recording of on-time drug pick-up and electronic monitors of adherence [3–7]. This latter might consist of a device that enables clinic staff to read out the adherence history of the patient since the last clinic visit [3,4], or perhaps even monitoring of adherence in real time using mobile phone technology [3, 8, 9]. Real time monitoring means there is the potential to react rapidly to missed doses to prevent default from care and reduce the risk of resistance development. Adherence monitoring-based interventions could be used to enable differentiation of care so that those with high adherence can have reduced visit frequency and/or attend for pharmacy-only visits, allowing appreciable non-ART clinic cost savings, as has been proposed based on viral load monitoring [10, 11]. In addition, even where viral load monitoring is in place, adherence monitoring-based interventions have potential to replace viral load measurement in people in whom viral suppression has been demonstrated.

Measured outcomes for studies of adherence monitoring-based interventions may include percent of doses taken (often referred to in the drug adherence literature as execution), duration of treatment (persistence), frequency of ART interruption, and the proportion of people with viral load suppression. However, the ultimate impact that adoption of such interventions would have on key program outcomes such as death rates and disability-adjusted life years (DALYs) averted is often unclear. Further, it is not intuitively clear how much money it is worth spending on an adherence monitoring-based intervention in order for its introduction to be cost-effective; i.e. of net health benefit given the opportunity costs of its introduction. In thinking of designing such interventions it would be useful to understand the approximate upper limit of cost that could be considered for an intervention of a given effectiveness, in order for the intervention to be cost effective.

In this paper we apply a model which captures the joint effects of adherence and viral resistance on response to ART in order to provide a link between viral suppression outcomes and these key program outcomes. This should facilitate use of data from trials of adherence monitoring-based interventions to be used to predict the population impact of the interventions, and hence allow assessment of their cost effectiveness.

## Methods

We used the HIV Synthesis model—an individual based model that models HIV sexual transmission, progression and the effect of ART in adults that models ART effects through adherence, accounting for current resistance to the ART regimen [11–14]. Variables simulated include the current average adherence (a 3 month average, but see below) for those on ART, viral load and CD4 count. Details of the assumptions in modelling of adherence are presented in section 6 of S1 File which provides full model details, but brief details are given here.

### Modelling of adherence and its effect on viral load and drug resistance outcomes

We refer to *adherence* specifically as the average adherence in people on antiretroviral drugs. Since our model updates in 3 month periods, short term interruptions of days or a few weeks are treated as sub-optimal average adherence during the 3 month period. Interruption of ART over periods of 3 months or greater are referred to as *ART discontinuation* and modelled explicitly. ART discontinuation is usually concomitant with disengagement from clinic attendance.

**Fig 1:**
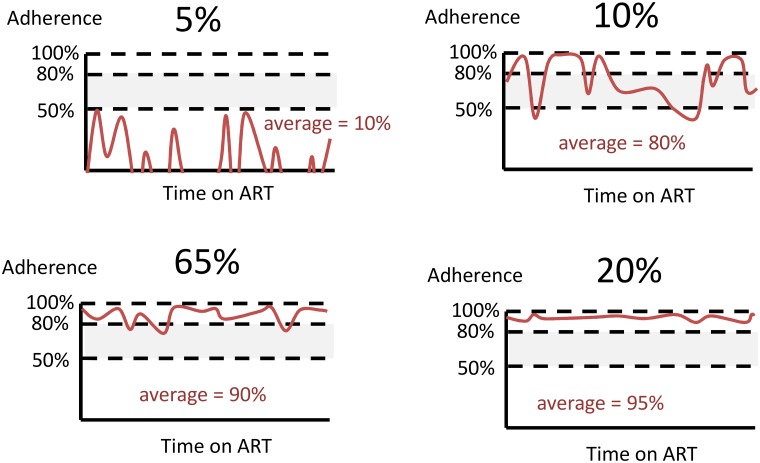
Modelling of distribution of adherence patterns over individuals in the population.

Average adherence in each 3 month period for an individual is determined from the underlying tendency to adhere (which is a lifelong value for the individual, unless changed as a result of an adherence intervention) with within-person period-to-period variability (Fig 1) [6, 15–24]. Each patient thus has a certain higher or lower tendency to adhere but their actual adherence varies over time, both at random and according to the presence of symptoms (with drug toxicity or presence of WHO stage 4 disease leading to a decrease in adherence) and age (tendency for increasing adherence with age).

The average adherence in each 3 month period is a value between 0%-100%. Effects of adherence on viral load and resistance acquisition risk are modelled by classifying levels into < 50%, 50–79%, ≥ 80%, with effects of ART on viral load suppression being greater the higher the adherence level and the resistance acquisition risk being highest in the 50%-79% category. We do not distinguish between patterns of adherence at a level more granular than the 3 monthly average level and hence cannot explicitly take into account the specific pattern within the 3 month period, which could be important (e.g. whether 80% adherence consists of missing drug one day in every five or a 1 week interruption in every 5 weeks). Thus the adherence level in each period should be conceived of as conveying the degree to which the pattern of adherence means that drug levels are maintained at intended therapeutic levels, rather than simply the average adherence over the period.

This distribution of adherence levels is primarily determined by the adherence levels required for the model outputs to mimic observed data. This includes data on rates of resistance development and virologic failure and also data on the proportion of patients at first virologic failure who have no resistance mutations present [25–37]. It is clear from such data in more recent years that the great majority of patients who started ART with 3 or more drugs are sufficiently adherent that virologic failure rates are low (and so resistance accumulation is also likely to be low) [17, 38].

The distribution of adherence over the first year of ART has been compared with data from a large programme in Zambia (see Fig K in S1 File; [39]). The degree to which outputs on viral load at one year from start of ART correspond to observed data is shown in Fig L in S1 File. These are reconstructed outcomes for all people who have initiated ART in Zimbabwe up to 2014 (the overall mean CD4 count at initiation is 145 /mm^3^). Figs M and N in S1 File compare Kaplan Meier estimates of time to virologic failure and resistance, respectively, between the model and observed data, in the latter case data are from the UK due to the lack of data from sub-Saharan Africa. Fig O in S1 File illustrates the proportion of people with resistance (amongst those on ART with non-suppressed viral load) and corresponds to estimates from the large WHO resistance surveillance.

### Discontinuation of ART

Within the model, people can simultaneously discontinue ART and disengage from care (i.e. they do not attend the clinic anymore) or can discontinue ART but still attend clinical visits. The basic rate of discontinuation (for reasons apart from drug stock-out) is assumed to be 0.02 per 3 months; this rate is doubled in people with current toxicity (note that in addition to this increased risk of discontinuation with current toxicity, there is assumed to be some substitution of drugs causing toxicity with available alternatives and a greater rate of discontinuation in patients with a greater tendency to be non-adherent (1.5-fold if the person’s adherence average is 50–79% and 2-fold if their adherence average is < 50%) [40]. In a systematic review, drug toxicity, adverse events and side effects have been found to be the most commonly given reasons for drug discontinuation [40].

The rate of discontinuation also reduces with time on ART, decreasing after 1 year [41–43]. If the person’s average adherence is > 80% then there is a 30% chance that discontinuation coincides with stopping visits to the clinic, if the long term average adherence is 50%-80% then there is a 45% chance, and if the long term average adherence is < 50% then there is a 60% chance. This is due to an assumption that factors leading to poor adherence are also likely to be associated with disengagement from care. The rate of discontinuation and disengagement from care is likely to vary by setting. Fig P in S1 File shows a comparison between modelled and observed (from a study by Kranzer et al. [42] of Kaplan Meier estimates of the percent of people having discontinued ART by time from ART initiation. Discontinuation of ART leads to return of viral load to the pre-ART maximum and a relatively rapid CD4 return of CD4 towards pre-ART value [44].

### Setting and intervention targeting and effect

We assume the adherence monitoring intervention is introduced in 2017 in the example setting of Zimbabwe. The effect of any given adherence intervention (Table 1) is modelled as effects on one or more of the following three elements: (i) e*nhanced average adherence when on ART—*implemented as a change in adherence to 95% for individuals in whom intervention is currently effective; (ii) r*educed risk of ART discontinuation—*implemented as a 75% reduction in risk of discontinuation for individuals in whom intervention is currently effective; (iii) r*educed risk of resistance acquisition—*implemented as a 50% lower risk of resistance for individuals in whom intervention is currently effective. In each case this represents a large effect at the individual level—the overall effect of the intervention is then determined by the percent of people in whom the intervention is effective. We consider scenarios in which the adherence intervention is effective in 20%, 50% or 80% of people to whom it is applied. We consider that the intervention has potential effects for all people on ART, including those who have high average adherence (e.g. by potentially reducing the risk of discontinuation). The intervention effect is considered to be durable for up to the full 20 year time horizon that we consider, although a shorter duration of effect (1 year) is considered in sensitivity analyses.

**Table 1:** Modelled intervention effects in individuals in whom adherence monitoring intervention is currently effective. In each case, this represents a large effect at the individual level—the overall effect of the intervention is then determined by the percent of people in whom the intervention is effective.

Parameter potentially influenced by adherence intervention	Value for parameter in absence of intervention	Intervention effect
Distribution of average adherence when on ART (each person’s underling tendency to adhere) * and see Fig 1.	5% of the population have long term average adherence 10%, period-to-period variability (standard deviation = 20%) 10% of the population have long term average adherence 80%, period-to-period variability (standard deviation = 20%) 65% of the population have long term average adherence 90%, period-to-period variability (standard deviation = 5%) 20% of the population have long term average adherence 95%, period-to-period variability (standard deviation = 5%)	Change in adherence to 95% for individuals in whom intervention is currently effective.
Risk of ART discontinuation	Rate of 0.02 per 3 months (with higher rate in those with ART toxicity and those with lower underlying tendency to adhere)	75% decline in rate of risk of discontinuation (to 0.005) per 3 months for individuals in whom intervention is currently effective.
Risk of resistance acquisition	Dependent on current adherence, current number of active drugs, current viral load (Details in appendix)	50% lower risk of resistance for individuals in whom intervention is currently effective.

* adherence in each 3 month period is determined by the person’s underlying tendency to adhere (which is a lifelong value, unless changed as a result of an adherence intervention) with within-person period-to-period variability. The within person variability is also a lifelong value. Adherence is also influenced by (i) current toxicity (current ADC) (ii) start second line (iii) viral load measurement > 1000 copies/mL. This distribution of adherence patterns is chosen on the basis of data on adherence and on patterns on virologic failure in people followed on ART. Details in S1 File.

### ART monitoring strategy

We consider two main scenarios regarding laboratory monitoring of people on ART. One in which the roll-out of viral load testing will occur as planned in Zimbabwe by 2017, the other in which viral load testing is not available and hence the CD4 count is still used (using a switching strategy based on absolute CD4 count) [11]. In the scenario in which we assume that viral load monitoring is in place we consider also the effects of viral load measurement triggering a positive effect on adherence. Viral load being identified as being above 1000 copies/mL is assumed to lead to an increase in adherence in 70% of people as a result of targeted adherence intervention; this is consistent with data showing that a high proportion of people with measured viral load > 1000 copies/mL who undergo an adherence intervention subsequently achieve viral suppression without a change in ART [45–48] and broadly consistent with a meta-analysis [49]. Although the appropriate duration to assume for this effect is uncertain [47] we assume, consistent with our previous work [11], that the adherence intervention is effective only the first time the viral load triggers an adherence intervention and that for 40% the effect is permanent (i.e. 70% x 40% = 28% of those with a viral load >1000), but that in the remaining 60% (i.e. 70% x 60% = 42% of those with viral load>1000) it lasts only 6 months.

### Differentiation of care

In the scenario in which viral load monitoring is not available (as remains the case in many low income settings in sub-Saharan Africa) we consider that the adherence monitoring-based intervention can be used to differentiate care. In particular, for those with high adherence over the past 3 month period, visits are simplified such that visit costs are reduced by 50% (from $20 per 3 months to $10), based on the concept that adherence can be rapidly checked and, if high and no symptoms present, then a pharmacy-only visit is possible. In terms of health outcomes, being on such a simplified visit schedule is assumed to have the effect that if a WHO stage 4 condition develops then the risk of death due to the condition is 1.5 times raised, as result of potentially later detection of symptoms by clinic staff. If viral load monitoring is used, then we assume that care can also be differentiated, primarily according to the most recent viral load measured in the past year, with again a 50% reduction in visit costs if the most recent viral load is < 1000 copies/mL and this has been measured in the past year. However, if the adherence monitoring-based intervention is in use and no viral load measure is available in the past 1 year but if the most recent viral load was < 1000 copies/mL and the adherence measure shows > 80% adherence then we again assume that visit costs are also reduced in this circumstance. This use of the objective adherence monitoring to identify additional opportunities for reduced clinic visits includes the second 3 month period after start of ART (because the first viral load test is at 6 months). In a key additional analysis we also consider the possibility that even when viral load monitoring is available the viral load testing will cease in those in whom viral load suppression has been demonstrated where there is an adherence monitoring based intervention in place and adherence is > 80%.

### Economic considerations

Programme costs resulting from adherence monitoring interventions are also considered to allow a full economic evaluation. The cost of the intervention is considered per person year on ART. Our objective is to maximize population health from within available health care resources. A health sector perspective has therefore been adopted for the analysis, so direct and indirect costs incurred by the patients are not included. Health benefits associated with the adherence intervention are estimated using the metric *DALYs averted* in the entire adult population. The increment in programme costs resulting from introduction of the adherence monitoring intervention divided by number of DALYs averted (the health benefit) gives the incremental cost effectiveness ratio (ICER). The ICER can be compared with a cost effectiveness threshold to ascertain whether the intervention is likely to represent an appropriate use of resources given the opportunity costs of using those resources for this purpose (i.e. to be cost effective). The cost-effectiveness threshold for a country represents the opportunity costs of resources required to fund the intervention, in terms of the health gains those resources could generate if used for alternative purposes in the public health care system [50]. As such, the threshold for a country is not readily apparent, but $500 per DALY averted is likely to be at the upper end based on the magnitude of benefit if resources were spent on other programmatic priorities [51]. We consider the cost that the intervention would have to be delivered at in order for it to be cost effective based on the $500 threshold. We consider a 20 year time perspective from 2017–2036. Both costs and health benefits were discounted to present value using a 3% per annum discount rate in our base case. The modelling results are intended to inform decisions in sub-Saharan African countries classified as low and low-middle income countries using the World Bank country classification.

Disability weights to calculate DALYs averted were derived from a recent comprehensive study [52]. Unit costs (in $US at 2014 prices) are detailed in S1 File. In brief, costs of viral load assays are assumed to be $22, counting all components of the cost (reagents, costs of equipment, human resources, buildings, etc.) (details in S1 File [53, 54]). Likewise, the cost of measuring CD4 counts is assumed to be $10 [55]. The current annual cost (including supply chain) of the first-line regimen of efavirenz, emtricitabine, tenofovir (assumed used as a fixed dose combination) is assumed to be $144 per person per year and second-line regimen of zidovudine, emtricitabine, ritonavir-boosted atazanavir $312 per person per year [56]. Programme costs for clinic visits (not including drug or viral load / CD4 count tests) are $20 per 3 months [57, 58] with an assumed reduction to $10 per 3 months when care is differentiated and the person has viral suppression or high measured adherence.

### Sensitivity analyses

Besides considering the various possible component effects of the adherence monitoring intervention, and varying the percent of people in whom the intervention has an effect, we considered the effect of varying several other factors. We considered scenarios in which: the overall population adherence profile was lower or higher; the background rate of treatment discontinuation was lower or higher; the intervention effect was of 1 year duration only; there was no differentiation of care based on adherence; there is no effect of viral load > 1000 on adherence; there was no differentiated care (neither driven by viral load nor by adherence); there is only a $5 per 3 month saving from adherence-informed differentiated care; and where the adherence intervention is used instead of viral load monitoring. Finally, we consider a sensitivity analysis in which we change the underlying adherence structure such that a person’s underlying tendency to adhere can shift during their lifetime for other reasons besides the adherence interventions, for example due to changes in life circumstances (their “lifetime” adherence is re-sampled from the original distribution used to generate each individuals lifetime adherence level). We operationlise this as a 2% chance per 3 month period that a person’s underlying tendency to adhere is changed, to a value based on a re-sampling from the distribution in Fig 1.

## Results

The status of the simulated adult population of Zimbabwe in 2014 is shown in Table A in S1 File. Outputs from the model include: number of adults living with HIV 1,161,000 out of a population size of 8,117,000 aged 15–65; prevalence of HIV in women age 15–45 17%; 2.8 million HIV tests done in the year; 655,000 people on ART, meaning that 56% of all people with HIV were on ART; death rate for people on ART 3.21 /100 person years, for all HIV positive people 5.22 /100 person years; of people on ART the proportion with viral load < 500 copies/mL 82%. By 2016, the model has 776,000 on ART (66% of all people with HIV).

The modelled outcomes of the introduction of an adherence monitoring intervention in 2017, according to the attributes of that intervention, are shown in Table 2. Outcomes shown are averages over 20 years from 2017–2036. As our main overall measure of the effect of the adherence monitoring-based interventions we consider the population of ART experienced people (i.e. i.e. all those who have ever started ART regardless of whether they remain on ART) and focus on the proportion of this population with high adherence and the proportion with viral load < 1000 copies / mL. The reason for considering this measure is that each of the various components of the effect of the adherence monitoring-based intervention act to increase this proportion. When considering our measure of population health, DALYs, we consider the whole adult population, those with and HIV and those without. This is because we wish to take into account the effects of the intervention on reduced HIV transmission. First (Table 2a), viral load monitoring is assumed not to be in place during the 20 year period (which is not likely to be the case in Zimbabwe itself but could be elsewhere in the region). If the intervention is effective in only 20% of the population then the effect on the proportion of ART experienced people with viral load suppression below 1000 copies/mL is only 2.7%, even when all three components of the effect are present: improving adherence in those on ART, reducing the rate of discontinuation of those on ART, and reducing the risk of resistance acquisition. This modest effect is largely due to the relatively high levels of adherence without the intervention. Even so, an intervention with this effect costing $37 per person on ART per year (including patients in whom the intervention is not effective) would be cost effective, due mainly to the clinic visit savings from differentiation of care. Considering that the intervention is effective in 50% of people rather than 20% there is now a 6.5% benefit on the proportion of ART experienced people with viral load suppression below 1000 copies/mL with the adherence intervention if it has an effect on all three components listed above. Of the three components, it is the effect on reducing the rate of ART discontinuation that had the greatest beneficial effects. The death rate in those with diagnosed HIV is likewise reduced, from 4.69 per 100 person years with no intervention to 4.08 per 100 person years (i.e. a 13% relative reduction) with an intervention having an effect on all three components. When the intervention has an effect on 80% of people then with an effect on all three components there is an 8.2% benefit in terms of proportion of ART experienced people with viral load suppression and a 20% reduction in death rate in people with diagnosed HIV from 4.69 to 3.73 / 100 person years. In this situation, an intervention with this effect costing $62 per person on ART per year would be cost effective. The maximum intervention cost to be cost-effective is lowest when the intervention only influences ART discontinuation (combination of components (c), second column in Table 2). This is due to the fact that more drug costs are incurred, whereas when there are beneficial effects on average adherence or on resistance acquisition while on ART there are no additional drug costs assumed.

**Table 2:** Outcomes of adherence interventions according to the components the intervention has an effect on, the percent of people the intervention is effective in and whether viral load monitoring is employed, all in the context of an intervention with a durable effect.

% of people adherence intervention effective in	Components intervention has beneficial effect on (a) adherence on ART only (b) adherence on ART + discontinuation of ART (c) discontinuation of ART only (d) adherence on ART + risk of resistance (e) adherence on ART + discontinuation of ART + risk of resistance	Proportion of ART experienced people on ART with high adherence	Proportion of ART experienced people with viral load < 1000	Death rate in ART experienced people	DALYs averted per 3 months (over 20 years, compared with no intervention)	Maximum cost of adherence intervention (per person on ART per year) to be cost effective
**(a) No viral load monitoring**						
No intervention	---	0.805	0.741	4.69	---	---
20	(a)	0.812	0.747	4.62	1226	$33
20	(b)	0.828	0.760	4.48	2889	$35
20	(c)	0.818	0.752	4.55	2203	$30
20	(d)	0.812	0.753	4.59	1534	$34
20	(e)	0.828	0.768	4.44	3483	$37
50	(a)	0.823	0.756	4.54	2425	$38
50	(b)	0.861	0.788	4.16	7409	$46
50	(c)	0.838	0.770	4.34	4822	$34
50	(d)	0.823	0.771	4.43	3562	$42
50	(e)	0.862	0.806	4.08	8282	$50
80	(a)	0.833	0.764	4.42	3868	$44
80	(b)	0.892	0.813	3.87	11836	$53
80	(c)	0.856	0.784	4.14	7445	$36
80	(d)	0.834	0.788	4.27	5901	$53
80	(e)	0.893	0.843	3.73	13017	$62
**(b) Viral load monitoring**						
No intervention	---	0.817	0.766	4.61	---	
20	(a)	0.823	0.772	4.56	1281	$8
20	(b)	0.838	0.784	4.42	2457	$8
20	(c)	0.830	0.778	4.48	2416	$7
20	(d)	0.823	0.775	4.53	1087	$7
20	(e)	0.839	0.790	4.37	3407	$12
50	(a)	0.831	0.778	4.47	2118	$11
50	(b)	0.867	0.811	4.13	6573	$17
50	(c)	0.848	0.795	4.26	4489	$8
50	(d)	0.832	0.791	4.40	2989	$17
50	(e)	0.868	0.825	4.04	7833	$23
80	(a)	0.840	0.785	4.41	3232	$17
80	(b)	0.895	0.835	3.83	11209	$28
80	(c)	0.867	0.811	4.03	7647	$12
80	(d)	0.840	0.805	4.25	5074	$26
80	(e)	0.896	0.859	3.70	12444	$36

**Fig 2:**
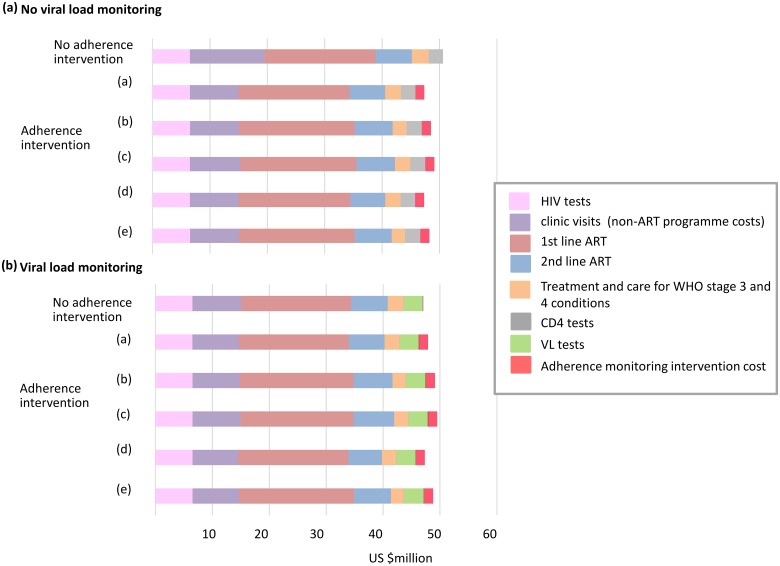
Overall programme costs according to components intervention has beneficial effect on ((a) adherence on ART only (b) adherence on ART + discontinuation of ART (c) discontinuation of ART only (d) adherence on ART + risk of resistance (e) adherence on ART + discontinuation of ART + risk of resistance). Costs in US$m per 3 months (mean 2017–2036, discounted at 3% per annum from 2015). For scenario with 50% of people in whom the intervention has an effect, and a durable effect. For illustrative purposes, the adherence intervention has an arbitrarily selected cost of $10 per year per person on ART.

Fig 2a shows the breakdown in costs. This is based on a notional intervention cost per year per person on ART (i.e. those adherent as well as those not adherent) of $10. The effect of the adherence monitoring based intervention in reducing costs can be seen. The component of the intervention that generally has the greatest beneficial effect on averting DALYs is to reduce the rate of discontinuation. This however involves greater use of ART and so tends to also increase costs. Improvements in ART adherence or risk of resistance in those already on ART has a more modest beneficial effect than an effect in reducing ART discontinuation but does not incur additional costs apart from the cost of the intervention and in fact leads to less use of second line ART which acts to relieve cost burden somewhat.

In sensitivity analyses (Table 3a), in which we consider that the intervention is effective in 50% of people, the intervention cost per person on ART per year required for it to be cost effective was in the $29-$58 range, except in the situation where we assume that care is not differentiated and thus there are no savings in visit costs ($5-$20 cost in order to be cost effective). Since the cost effectiveness is mainly dependent on the ability of adherence monitoring to differentiate care, the intervention is cost effective in the range $30-$35 per person on ART per year even where the effect of the intervention in improving adherence lasts only 1 year.

**Table 3:** Sensitivity analyses (a) in context of no viral load monitoring (b) in context of viral load monitoring. All in context of an intervention with a durable effect and the intervention being effective in 50% of people.

**(a) no viral load monitoring**					
Sensitivity analysis	Components intervention has beneficial effect on	Proportion of ART experienced people with viral load < 1000	Death rate in ART experienced people	DALYs averted per 3 months (over 20 years, compared with no intervention)	Maximum cost of adherence intervention (per person on ART per year) to be cost effective
Base case	No intervention	0.741	4.69	---	
	(a)	0.756	4.54	2425	$38
	(b)	0.788	4.16	7409	$46
	(c)	0.770	4.34	4822	$34
	(d)	0.771	4.43	3562	$42
	(e)	0.806	4.08	8282	$50
Lower adherence	No intervention	0.651	5.85	.	
	(a)	0.699	5.20	9977	$64
	(b)	0.733	4.76	15105	$68
	(c)	0.673	5.46	4779	$28
	(d)	0.714	5.11	10601	$69
	(e)	0.753	4.67	16382	$76
Higher adherence	No intervention	0.768	4.40	.	
	(a)	0.772	4.35	101	$29
	(b)	0.802	4.00	5246	$37
	(c)	0.796	4.07	4035	$33
	(d)	0.788	4.24	2125	$38
	(e)	0.821	3.91	6696	$45
Lower rate of ART discontinuation	No intervention	0.778	4.21	.	
	(a)	0.795	4.05	2654	$39
	(b)	0.812	3.86	5400	$42
	(c)	0.792	4.04	2135	$29
	(d)	0.812	3.94	3378	$42
	(e)	0.83	3.77	6324	$48
Higher rate of ART discontinuation	No intervention	0.672	5.65	.	
	(a)	0.683	5.47	2328	$36
	(b)	0.744	4.73	11744	$50
	(c)	0.725	4.92	9384	$39
	(d)	0.696	5.37	3688	$43
	(e)	0.761	4.64	13117	$58
Duration of effect 1 year only	No intervention	0.741	4.69	.	
	(a)	0.744	4.66	802	$31
	(b)	0.746	4.61	2026	$34
	(c)	0.744	4.65	1125	$30
	(d)	0.747	4.63	1172	$33
	(e)	0.750	4.59	2328	$35
No adherence-informed differentiated care	No intervention	0.741	4.70	.	
	(a)	0.755	4.53	2205	$8
	(b)	0.787	4.15	7414	$15
	(c)	0.768	4.34	4812	$5
	(d)	0.771	4.43	3228	$12
	(e)	0.806	4.06	8417	$20
Alternative underlying adherence pattern	No intervention	0.719	4.87	.	
	(a)	0.743	4.62	3467	$40
	(b)	0.776	4.25	7815	$45
	(c)	0.745	4.51	4608	$31
	(d)	0.759	4.55	4017	$45
	(e)	0.795	4.15	9448	$54
**(b) viral load monitoring**					
Sensitivity analysis	Components intervention has beneficial effect on	Proportion of ART experienced people with viral load < 1000	Death rate in ART experienced people	DALYs averted over 20 years (compared with no intervention)	Maximum cost of adherence intervention (per person on ART per year) to be cost effective
Base case	No intervention	0.766	4.61	---	
	(a)	0.778	4.47	2118	$11
	(b)	0.811	4.13	6573	$17
	(c)	0.795	4.26	4489	$8
	(d)	0.791	4.40	2989	$17
	(e)	0.825	4.04	7833	$23
Viral load testing stopped for people with viral load < 1000 if adherence > 80%	No intervention	0.766	4.61	.	
	(a)	0.772	4.56	1381	$19
	(b)	0.805	4.21	6242	$27
	(c)	0.789	4.34	3963	$16
	(d)	0.786	4.44	2360	$25
	(e)	0.821	4.09	7303	$32
Lower adherence	No intervention	0.689	5.51	.	
	(a)	0.729	5.04	7296	$35
	(b)	0.765	4.62	12411	$39
	(c)	0.713	5.13	4913	$7
	(d)	0.743	4.97	8701	$42
	(e)	0.780	4.54	13849	$47
Higher adherence	No intervention	0.789	4.36	.	
	(a)	0.793	4.32	1099	$6
	(b)	0.823	3.99	4989	$12
	(c)	0.819	4.04	3702	$6
	(d)	0.805	4.23	1787	$11
	(e)	0.838	3.90	6721	$20
Lower rate of ART discontinuation	No intervention	0.806	4.12	.	
	(a)	0.819	4.00	2173	$11
	(b)	0.837	3.81	4073	$12
	(c)	0.820	3.97	2049	$3
	(d)	0.834	3.92	2778	$16
	(e)	0.852	3.74	5265	$19
Higher rate of ART discontinuation	No intervention	0.694	5.56	.	
	(a)	0.703	5.44	1470	$9
	(b)	0.764	4.70	10577	$23
	(c)	0.748	4.87	8692	$15
	(d)	0.714	5.33	2838	$16
	(e)	0.778	4.62	11739	$31
Duration of effect 1 year only	No intervention	0.766	4.61	---	
	(a)	0.768	4.59	572	$5
	(b)	0.769	4.55	1853	$8
	(c)	0.767	4.58	1111	$5
	(d)	0.770	4.56	1175	$7
	(e)	0.771	4.51	2146	$9
No adherence-informed differentiated care	No intervention	0.766	4.60	.	
	(a)	0.777	4.48	1804	$8
	(b)	0.81	4.13	6618	$18
	(c)	0.795	4.25	4744	$7
	(d)	0.791	4.38	2818	$13
	(e)	0.825	4.02	7683	$21
No effect of viral load > 1000 on adherence	No intervention	0.759	4.69	.	
	(a)	0.772	4.53	2422	$13
	(b)	0.807	4.16	7078	$18
	(c)	0.79	4.33	4806	$9
	(d)	0.785	4.44	3419	$18
	(e)	0.822	4.08	8519	$26
No differentiated care (neither driven by viral load nor by adherence)	No intervention	0.765	4.61	.	
	(a)	0.777	4.47	1794	$7
	(b)	0.811	4.10	6561	$12
	(c)	0.795	4.24	4410	$4
	(d)	0.79	4.38	2532	$11
	(e)	0.825	4.03	7694	$19
Adherence intervention instead of viral load monitoring **	No intervention	0.766	4.60	.	
	(a)	0.756	4.54	930	$10
	(b)	0.788	4.17	5760	$18
	(c)	0.768	4.34	3224	$5
	(d)	0.771	4.43	2018	$16
	(e)	0.806	4.07	6869	$23
Alternative underlying adherence pattern	No intervention	0.747	4.75	.	
	(a)	0.767	4.56	2775	$15
	(b)	0.801	4.19	7777	$23
	(c)	0.775	4.39	4410	$6
	(d)	0.78	4.49	3397	$20
	(e)	0.816	4.12	8480	$28

** here only the no intervention scenario has viral load monitoring.

When viral load monitoring is available (Table 2b), benefits in terms of viral suppression, death rates and DALYs are similar to the case without viral load monitoring. However, since there is less of a benefit in cost due to the fact that care is already differentiated, based on viral load (Fig 2), the cost of the adherence monitoring-based intervention required to be cost effective is substantially lower. Still, an intervention costing $23 per year per person on ART, if it increases the proportion of ART experienced people with viral load < 1000 cps/mL by 5.9%, would be cost effective.

If viral load monitoring is available as well as an adherence monitoring intervention then perhaps a more likely situation than our base case is that viral load measurement will be ceased in people with a previous viral load level measured below 1000 copies/mL and > 80% adherence since the viral load measurement. In this case (Table 3b), an intervention costing $32 per year per person on ART, if it increases the proportion of ART experienced people with viral load < 1000 cps/mL by 5.5%, would be cost effective.

In sensitivity analyses (Table 3b), the maximum intervention cost permissible for it to be cost effective is also higher in the situations where the adherence levels in the population are generally lower or when the intervention replaces viral load monitoring rather than being in addition.

## Discussion

In this paper we use a mathematical model which captures the joint effects of adherence and viral resistance on response to ART in order to provide a link between viral suppression outcomes of a putative adherence monitoring intervention and key programme outcomes of death rates and DALYs. This should facilitate use of data from trials of adherence monitoring interventions to be used to predict the population impact of the interventions, and hence allow assessment of cost effectiveness of such interventions. Our results should also be useful in the design of trials when specifying the target effect for viral load or adherence outcomes, for sample size calculation.

In assessing cost effectiveness of adherence monitoring-based interventions it is important to consider what laboratory monitoring is in place, if any. We considered our adherence monitoring intervention in three main contexts: one in the presence of viral load monitoring where viral load monitoring continues alongside the adherence monitoring intervention, one in which viral load monitoring is available but is selectively replaced by adherence monitoring, and one in the complete absence of viral load monitoring. Given that the non-ART programme costs of providing for clinic visits average $60-$90 per year in low income settings in Africa, approaches that enable differentiation of care to allow less clinical visits in those doing well on ART are potentially highly significant [58]. We assumed that in the scenario where viral load monitoring is available such an approach is employed. However, although viral load monitoring is recommended in WHO guidelines, the roll out in many countries in Africa towards that goal has been modest, largely due to the complexities of setting up and continually running central laboratories with the capacity to meet demand for measurement of viral load on dried blood spot or plasma samples, and the current lack of a suitable affordable point of care assay [53]. For this reason we also considered a scenario in which viral load monitoring is not available, and here we assumed a CD4 count monitoring strategy is used, without differentiation of care (in the absence of an adherence-monitoring intervention).

Adherence monitoring-based interventions also could potentially be used to enable differentiation of care so that those with high adherence can have reduced visit frequency, reduced VL monitoring and/or attend for pharmacy-only visits. This represents a significant potential benefit, alongside the beneficial effect of improvements in adherence/retention in those with sub-optimal adherence. In settings without viral load monitoring available, the potential for adherence monitoring based interventions to represent a cost-effective approach is substantial. Our results suggest that adherence monitoring-based interventions can cost as much as approximately $62 per person on ART per year and represent a cost effective approach. This is an average cost over all people on ART, including those who are adherent without any intervention. Considering that costs for the approximately 80% of people with high adherence might be no more than $5 per year, this leaves $290 per year each for the approximately 20% of people who have suboptimal or poor adherence. Even where viral load monitoring is available there is a potentially important role for adherence monitoring-based interventions. We assumed in our base case that care would be differentiated according to the adherence level, measured by adherence monitoring, whenever the most recent viral measure was < 1000 cps/mL but more than one year in the past. With an adherence monitoring based intervention leading to a 5% increase in the proportion of ART experienced people with viral suppression < 1000 copies/mL (Table 2) the intervention is cost effective if costing $23 per year person on ART or less ($95 per year for each of the 20% with poor adherence). An alternative, and perhaps more likely, approach could be to stop viral load monitoring in those with high levels of adherence and continue on a reduced visit frequency unless the adherence level declines, at which point viral load monitoring can be resumed until viral load suppression and high adherence are achieved. With this approach the adherence intervention can be as high as $32 per person on ART per year and remain cost effective. Further studies to compare health outcomes from different approaches to use adherence monitoring in tandem with viral load testing in a cost efficient way would be helpful. We assume a modest effect of viral load being measured above 1000 copies/mL on adherence, but this was not very influential in our results and the maximum cost for the intervention to be cost effective was similar in sensitivity analyses when this assumed beneficial effect on adherence was removed.

The relationship between the proportion of ART experienced people virally suppressed and mortality in ART experienced people will depend on various characteristics of the HIV positive population, particularly the CD4 count profile and the CD4 count nadir (the level to which the CD4 will tend to fall over the first few months of ART discontinuation, should this occur). Our findings are most specifically relevant to Zimbabwe, but given that we have considered a range of scenarios in terms of existing ART monitoring strategy (viral load monitoring or not), population adherence profile, and population tendency to discontinue ART, our findings regarding the translation of intervention effectiveness into DALYs averted should be reasonably generalizable across sub-Saharan Africa. Regarding costs, we provide a breakdown of cost components so that country-specific costs for other countries could be utilized if required. We use a cost-effectiveness threshold of $500 to reflect the value (opportunity costs) of other claims on resources, but this value is uncertain and may well be different in other countries. A lower threshold will mean that intervention costs need to be lower in order to be cost-effective.

Examples of objective adherence-monitoring based interventions are use of pharmacy and drug pick up records and use of electronic adherence monitors, including real time monitors using mobile-phone technology. Neither is a perfect measure but both are likely to be more accurate than subjective measures such as self report. Perhaps the most relevant objective measure of adherence is the plasma drug level but it is currently hard to conceive that this latter measure could be used in routine care.

To our knowledge there is little similar work to ours which has been conducted. Kessler and colleagues considered the impact and cost-effectiveness of hypothetical strategies to enhance retention in care within HIV treatment programs in East Africa [59]. They concluded that programs should consider retention-focused programs once they have already achieved high degrees of ART coverage among eligible patients, and noted that it is important that decision makers understand the epidemiology and associated outcomes of those patients who are classified as lost to follow up in their systems prior to implementation in order to achieve the highest value. In addition, Petersen et al, have used marginal structural models to estimate the effect of pillbox organisers on adherence and viral load outcomes and, in the United States context, found them to be associated with cost per QALY of $19,000 [60]. Cost effectiveness of adherence interventions has been reviewed [61], with the conclusion that they can be cost effective, but those results are of limited relevance for sub-Saharan Africa.

Converting the efficacy, measured in terms of level of viral load suppression, into clinical and mortality outcomes is not straightforward. This issue was initially raised when the Food and Drug Administration was taking a decision on whether to licence drugs based on trials with viral load rather than clinical endpoints as outcomes [62–64]. Analyses of trials in which viral load was assessed as well as clinical outcomes were used.

There are limitations of this work. The model updates in 3 month periods and therefore it is not suited to capture specific patterns of adherence. Because average adherence is a relatively insensitive predictor of VL failure compared to discontinuations, this is a conservative bias. Electronic monitoring which incorporates discontinuations would add to the predictive power, lead to better differentiation and improve cost effectiveness. Qualitative data by Ware and quantitative data by Haberer suggests that the mere presence of electronic monitoring creates a perception of “connectedness to clinic” and may improve adherence by the presence of monitoring alone, even without explicit intervention [65, 66]. Such a Hawthorne effect would increase the cost-effectiveness of our estimates. Lastly, by the nature of any model we have not captured all subtleties of real life and cannot rule out that some are important in affecting the outcomes we modelled. Lastly, we used a 20 year time horizon and ideally we would have used a longer time period to allow effects to play out over a longer period.

In conclusion, our results suggest that there is clear scope for adherence monitoring-based interventions to provide net population health gain in low income settings in sub-Saharan Africa, with potential cost-effective use in situations where viral load monitoring is or is not available. Our results should guide the implementation of future adherence monitoring interventions found in randomized trials and other studies to have health benefit.

## Supporting Information

S1 File (DOCX)

## References

[1] Bärnighausen T, Chaiyachati K, Chimbindi N, Peoples A, Haberer J, Newell M-L. Interventions to increase antiretroviral adherence in sub-Saharan Africa: a systematic review of evaluation studies. Lancet Infect Dis 2011; 11: 942–51. 10.1016/S1473-3099(11)70181-5 22030332 PMC4250825

[2] Chaiyachatia KH, Ogbuojib O, Price M, Suthar AB, Negussie EK, Bärnighausen T. Interventions to improve adherence to antiretroviral therapy: a rapid systematic review. AIDS 2014; 28 (Suppl 2):S187–S204 24849479 10.1097/QAD.0000000000000252

[3] Campbell JI, Haberer JE. Cell Phone-Based and Adherence Device Technologies for HIV Care and Treatment in Resource-Limited Settings: Recent Advances. Curr HIV/AIDS Rep (2015) 12:523–531. 26439917 10.1007/s11904-015-0282-8

[4] Demonceau J, Ruppar T, Kristanto P, Hughes DA, Fargher E, Kardas P et al Identification and Assessment of Adherence-Enhancing Interventions in Studies Assessing Medication Adherence Through Electronically Compiled Drug Dosing Histories: A Systematic Literature Review and Meta-Analysis. Drugs (2013) 73:545–562 10.1007/s40265-013-0041-3 23588595 PMC3647098

[5] Pellowski JA, Kalichman SC. Recent Advances (2011–2012) in Technology-Delivered Interventions for People Living with HIV. Curr HIV/AIDS Rep 2012; 9:326–334 10.1007/s11904-012-0133-9 22922945 PMC3492505

[6] Osterberg L. Blaschke T. Adherence to Medication. NEJM 2005; 353, 487–497 10.1056/NEJMra050100 16079372

[7] Blaschke TF, Osterberg L, Vrijens B, Urquhart J. Adherence to Medications: Insights Arising from Studies on the Unreliable Link Between Prescribed and Actual Drug Dosing Histories. Annu. Rev. Pharmacol. Toxicol. 2012 52:275–301. 10.1146/annurev-pharmtox-011711-113247 21942628

[8] Haberer J, Kiwanukad J, Nansera D, Muzoora C, Hunt PW, Soe J, et al Realtime adherence monitoring of antiretroviral therapy among HIV-infected adults and children in rural Uganda. AIDS 2013; 13:2166–2168. 10.1097/QAD.0b013e328363b53f PMC3868644 23751260

[9] Orrell C, Cohen K, Mauff K, Bangsberg DR, Maartens G, Wood R. A Randomized Controlled Trial of Real-Time Electronic Adherence Monitoring With Text Message Dosing Reminders in People Starting First-Line Antiretroviral Therapy. J Acquir Immune Defic Syndr 2015;70:495–502 10.1097/QAI.0000000000000770 26218411

[10] Duncombe C, Rosenblum S, Hellmann N, Holmes C, Wilkinson L, Biot M, et al Reframing HIV care: putting people at the centre of antiretroviral delivery. Trop Med Hygiene 2015; 20: 430–447 10.1111/tmi.12460 PMC4670701 25583302

[11] Working Group on Modelling of ART Monitoring Strategies in Sub-Saharan Africa. Sustainable HIV treatment in Africa through viral load-informed differentiated care. Nature 2015; 528:S68–76. 10.1038/nature16046 26633768 PMC4932825

[12] Phillips AN, Pillay D, Garnett G, Bennet D, Vitoria M, Cambiano V, et al Effect on transmission of HIV-1 resistance of timing of implementation of viral load monitoring to determine switches from first to second-line regimens in resource-limited settings. AIDS 2011; 25: 843–50. 10.1097/QAD.0b013e328344037a 21192233

[13] Cambiano V, Bertagnolio S, Jordan M, Pillay D, Perriens J, Venter F, et al Predicted levels of HIV drug resistance: potential impact of expanding diagnosis, retention, and eligibility criteria for antiretroviral therapy initiation. AIDS 2014, 28 (Suppl 1):S15–S23. 24468943 10.1097/QAD.0000000000000082

[14] Nakagawa F, Lodwick RK, Smith CJ, Smith R, Cambiano V, Lundgren JD, et al Projected life expectancy of people with HIV according to timing of diagnosis. AIDS 2012;26(3):335–43. 10.1097/QAD.0b013e32834dcec9 22089374

[15] Cambiano V, Lampe FC, Rodger AJ, Smith CJ, Geretti AM, Lodwick RK, et al Long-term trends in adherence to antiretroviral therapy from start of HAART. AIDS 2010 24, (8) 1153–1162 10.1097/QAD.0b013e32833847af 20299959

[16] Carrieri P, Cailleton V, Le M V, Spire B, Dellamonica P, Bouvet E, et al The dynamic of adherence to highly active antiretroviral therapy: results from the French National APROCO cohort. *J*.*Acquir*.*Immune*.*Defic*.*Syndr*., 2001 28, (3) 232–239 11694829 10.1097/00042560-200111010-00005

[17] El-Khatib Z, Ekstrom AM, Coovadia A, Abrams EJ, Petzold M, Katzenstein D, et al Adherence and virologic suppression during the first 24 weeks on antiretroviral therapy among women in Johannesburg South Africa—a prospective cohort study *BMCPublic Health* 2011; 11 88 10.1186/1471-2458-11-88 PMC3046911 21303548

[18] Genberg BL, Wilson IB, Bangsberg DR, Arnsten J, Goggin K, Remien RH, et al Patterns of antiretroviral therapy adherence and impact on HIV RNA among patients in North America *AIDS* 2012; 26 (11) 1415–1423 10.1097/QAD.0b013e328354bed6 22767342 PMC3655551

[19] Glass TR, Battegay M, Cavassini M, De GS, Furrer H, Vernazza PL, et al Longitudinal analysis of patterns and predictors of changes in self-reported adherence to antiretroviral therapy: Swiss HIV Cohort Study *JAcquirImmuneDeficSyndr* 2010; 54 (2) 197–203 10.1097/QAI.0b013e3181ca48bf 20035231

[20] Kleeberger CA, Buechner J, Palella F, Detels R, Riddler S, Godfrey R, et al Changes in adherence to highly active antiretroviral therapy medications in the Multicenter AIDS Cohort Study *AIDS* 2004; 18 (4) 683–688 15090774 10.1097/00002030-200403050-00013

[21] Lazo M, Gange SJ, Wilson TE, Anastos K, Ostrow DG, Witt MD, et al Patterns and predictors of changes in adherence to highly active antiretroviral therapy: longitudinal study of men and women *ClinInfectDis* 2007; 45 (10) 1377–1385 10.1086/522762 17968839

[22] Levine AJ, Hinkin CH, Castellon SA, Mason KI, Lam MN, Perkins A, et al Variations in patterns of highly active antiretroviral therapy (HAART) adherence AIDS Behav 2005; 9 (3) 355–362 10.1007/s10461-005-9009-y 16088365

[23] Mannheimer S, Friedland G, Matts J, Child C, Chesney M. The consistency of adherence to antiretroviral therapy predicts biologic outcomes for human immunodeficiency virus-infected persons in clinical trials *ClinInfectDis* 2002; 34 (8) 1115–1121 10.1086/339074 11915001

[24] Meresse M, March L, Kouanfack C, Bonono RC, Boyer S, Laborde-Balen G, et al Patterns of adherence to antiretroviral therapy and HIV drug resistance over time in the Stratall ANRS 12110/ESTHER trial in Cameroon *HIVMed* 2014 10.1111/hiv.12140 24589279

[25] Fox MP, van Cutsem G, Giddy J, Maskew M, Keiser O, Prozesky H, et al Rates and predictors of failure of first-line antiretroviral therapy and switch to second-line ART in South Africa JAcquirImmuneDeficSyndr 2012; 60 (4) 428–437 10.1097/QAI.0b013e3182557785 PMC3392418 22433846

[26] Bangsberg DR, Moss AR, Deeks SG. Paradoxes of adherence and drug resistance to HIV antiretroviral therapy. J Antimicrob Chem 2004; 53 (5): 696–699. 10.1093/jac/dkh162 15044425

[27] Bangsberg DR, Acosta EP, Gupta R, Guzman D, Riley ED, Harrigan PR, et al Adherence-resistance relationships for protease and non-nucleoside reverse transcriptase inhibitors explained by virological fitness *AIDS* 2006; 20 (2) 223–231 10.1097/01.aids.0000199825.34241.49 16511415

[28] Hamers RL, Wallis CL, Kityo C, Siwale M, Mandaliya K, Conradie F, et al HIV-1 drug resistance in antiretroviral-naive individuals in sub-Saharan Africa after rollout of antiretroviral therapy: a multicentre observational study Lancet InfectDis 2011; 11 (10) 750–759 10.1016/S1473-3099(11)70149-9 21802367

[29] Hassan AS, Nabwera HM, Mwaringa SM, Obonyo CA, Sanders EJ, Rinke de Wit TF, et al HIV-1 virologic failure and acquired drug resistance among first-line antiretroviral experienced adults at a rural HIV clinic in coastal Kenya: a cross-sectional study AIDS ResTher 2014; 11 (1) 9 10.1186/1742-6405-11-9 PMC3922732 24456757

[30] Hoffmann CJ, Charalambous S, Grant AD, Morris L, Churchyard GJ, Chaisson RE. Durable HIV RNA resuppression after virologic failure while remaining on a first-line regimen: a cohort study TropMedIntHealth 2014; 19 (2) 236–239 10.1111/tmi.12237 PMC4070658 24588012

[31] Kobin AB, Sheth NU. Levels of adherence required for virologic suppression among newer antiretroviral medications AnnPharmacother 2011; 45 (3) 372–379 10.1345/aph.1P587 21386024

[32] Li JZ, Gallien S, Ribaudo H, Heisey A, Bangsberg DR, Kuritzkes DR. Incomplete adherence to antiretroviral therapy is associated with higher levels of residual HIV-1 viremia AIDS 2014; 28 (2) 181–186 10.1097/QAD.0000000000000123 24361679 PMC4193963

[33] Mackie NE, Phillips AN, Kaye S, Booth C, Geretti AM. Antiretroviral drug resistance in HIV-1-infected patients with low-level viremia J Infect Dis 2010; 201 (9) 1303–1307 10.1086/651618 20350161

[34] Rosenblum M, Deeks SG, van der Laan M, Bangsberg DR. The risk of virologic failure decreases with duration of HIV suppression at greater than 50% adherence to antiretroviral therapy PloS One 2009 4 (9) e7196 10.1371/journal.pone.0007196 19787058 PMC2747009

[35] Tran DA. Wilson DP. Shakeshaft A. Ngo AD. Doran C. Zhang L. Determinants of virological failure after 1 year's antiretroviral therapy in Vietnamese people with HIV: findings from a retrospective cohort of 13 outpatient clinics in six provinces Sex Transm Infect 2014 10.1136/sextrans-2013-051353 24619575

[36] Usitalo A, Leister E, Tassiopoulos K, Allison S, Malee K, Paul ME, et al Relationship between viral load and self-report measures of medication adherence among youth with perinatal HIV infection AIDS Care 2014; 26 (1) 107–115 10.1080/09540121.2013.802280 23800360 PMC4190051

[37] von Wyl V, Klimkait T, Yerly S, Nicca D, Furrer H, Cavassini M, et al Adherence as a predictor of the development of class-specific resistance mutations: the Swiss HIV Cohort Study PloS One 2013; 8 (10) e77691 10.1371/journal.pone.0077691 24147057 PMC3797701

[38] Johannessen A, Naman E, Kivuyo SL, Kasubi MJ, Holberg-Petersen M, Matee MI, et al Virological efficacy and emergence of drug resistance in adults on antiretroviral treatment in rural Tanzania BMC Infect Dis 2009; 9 108 10.1186/1471-2334-9-108 PMC2713244 19583845

[39] Chi BH, Cantrell RA, Zulu I, Mulenga LB, Levy JW, Tambatamba BC, et al Adherence to first-line antiretroviral therapy affects non-virologic outcomes among patients on treatment for more than 12 months in Lusaka Zambia Int J Epidemiol 2009; 38 (3) 746–756 10.1093/ije/dyp004 19223334 PMC2689395

[40] Kranzer K, Ford N. Unstructured treatment interruption of antiretroviral therapy in clinical practice: a systematic review *TropMedIntHealth* 2011; 16 (10) 1297–1313 10.1111/j.1365-3156.2011.02828.x 21718394

[41] Kranzer K, Lewis JJ, Ford N, Zeinecker J, Orrell C, Lawn SD, et al Treatment interruption in a primary care antiretroviral therapy program in South Africa: cohort analysis of trends and risk factors J Acquir Immune Defic Syndr 2010; 55 (3) e17–e23 10.1097/QAI.0b013e3181f275fd 20827216 PMC3024539

[42] Tassie JM, Baijal P, Vitoria MA, Alisalad A, Crowley SP, Souteyrand Y. Trends in retention on antiretroviral therapy in national programs in low-income and middle-income countries J Acquir Immune Defic Syndr 2010; 54 (4) 437–441 10.1097/QAI.0b013e3181d73e1b 20351559

[43] Wandeler G, Keiser O, Pfeiffer K, Pestilli S, Fritz C, Labhardt ND, et al Outcomes of antiretroviral treatment programs in rural Southern Africa J Acquir Immune Defic Syndr 2012; 59 (2) e9–16 10.1097/QAI.0b013e31823edb6a 22067665 PMC3259205

[44] Grund B for the SMART Study Group. Predictors of initial CD4 decline after antiretroviral treatment interruption in the SMART study. XVI International AIDS Conference. August 13–18, 2006. Toronto. Abstract THPE0144.

[45] Orrell C, Harling G, Lawn SD, Kaplan R, McNally M, Bekker L-G, et al Conservation of first-line antiretroviral treatment regimen where therapeutic options are limited. Antiviral Therapy 2007; 12:83–88. 17503751

[46] Hoffmann CJ, Charalambous S, Sim J, et al Viremia, Resuppression, and Time to Resistance in Human Immunodeficiency Virus (HIV) Subtype C during First-Line Antiretroviral Therapy in South Africa. Clin Infect Dis 2009; 49:1928–35. 10.1086/648444 19911963 PMC2789416

[47] Hoffmann CJ, Charalambous S, Grant AD, Morris L, Churchyard GJ, Chaisson RE. Durable HIV RNA resuppression after virologic failure while remaining on a first-line regimen: a cohort study. Trop Med & Int Health 2013 10.1111/tmi.12237 PMC4070658 24588012

[48] Rutstein SE, Hosseinipour MC, Kamwendo D, Soko A, Mkandawire M, Biddle AK, et al Dried Blood Spots for Viral Load Monitoring in Malawi: Feasible and Effective. PLoS ONE 2015; 10(4): e0124748 10.1371/journal.pone.0124748 25898365 PMC4405546

[49] Bonner K, Mezochow A, Roberts T, Ford N, Cohn J. Viral load monitoring as a tool to reinforce adherence: a systematic review *JAcquirImmuneDeficSyndr* 2013; 64 (1) 74–78 10.1097/QAI.0b013e31829f05ac 23774877

[50] Claxton K, Walker S, Palmer S, Sculpher M. ‘Appropriate Perspectives for Health Care Decisions’ Centre for Health Economics Research Paper 54 University of York 2010

[51] Woods E, Revill P, Sculpher M, Claxton K. Country-Level Cost- Effectiveness Thresholds: Initial Estimates and the Need for Further Research. https://www.york.ac.uk/media/che/documents/papers/researchpapers/CHERP109_cost-effectiveness_threshold_LMICs.pdf 10.1016/j.jval.2016.02.017 PMC5193154 27987642

[52] Salomon JA, Vos T, Hogan DR, Gagnon M, Naghavi M, Mokdad A et al Common values in assessing health outcomes from disease and injury: disability weights measurement study for the Global Burden of Disease Study 2010. Lancet 2012; 380: 2129–43. 10.1016/S0140-6736(12)61680-8 23245605 PMC10782811

[53] Roberts T, Cohn J, Bonner K, Hargreaves S. Scale-up of Routine Viral Load Testing in Resource-Poor Settings: Current and Future Implementation Challenges. Clin Infect Dis 2016; 10.1093/cid/ciw001 PMC4803106 26743094

[54] Global Fund. Strategic reviews in sourcing and market dynamics. Available at: http://www.theglobalfund.org/en/p4i/events/. Accessed November 2015.

[55] Hyle EP, Jani IV, Lehe J, Su AE, Wood R, Quevedo J, et al The Clinical and Economic Impact of Point-of-Care CD4 Testing in Mozambique and Other Resource-Limited Settings: A Cost-Effectiveness Analysis. PLoS Med 2014, 11(9), e1001725 10.1371/journal.pmed.1001725 25225800 PMC4165752

[56] MSF. Untangling the web of antiretroviral price reductions. 17th Edition—July 2014. www.msfaccess.org.

[57] Siapka M, Remme M, Dayo Obure C, Maier C, Dehne KL, Vassall A. Is there scope for cost savings and efficiency gains in HIV services? A systematic review of the evidence from low- and middle-income countries. Bull World Health Organ 2014;92:499–511AD **|** 10.2471/BLT.13.127639 25110375 PMC4121865

[58] Tagar E, Sundaram M, Condliffe K, Matatiyo B, Chimbwandira F, Chilima B, et al Multi-Country Analysis of Treatment Costs for HIV/AIDS (MATCH): Facility-Level ART Unit Cost Analysis in Ethiopia, Malawi, Rwanda, South Africa and Zambia. PLoS ONE 2014; 9(11): e108304 10.1371/journal.pone.0108304 25389777 PMC4229087

[59] Kessler J, Nucifora K. Impact and Cost-Effectiveness of Hypothetical Strategies to Enhance Retention in Care within HIV Treatment Programs in East Africa. Value in Health 2015; 946–955 10.1016/j.jval.2015.09.2940 26686778 PMC4696404

[60] Petersen ML, Wang Y, van der Laan MJ, Guzman D, Riley E, Bangsberg DR. Pillbox organizers are associated with improved adherence to HIV antiretroviral therapy and viral suppression: A marginal structural model analysis. Clin Infect Dis 2007; 45: 908–915 10.1086/521250 17806060 PMC2442040

[61] Mathes T, Pieper D, Sunya-Lee A, Pieper Dawid, Eikermann M. Cost-effectiveness of adherence interventions for highly active ART: a systematic review. Int J Tech Ass in Health Care 2013; 29: 227–233 10.1017/S0266462313000317 23759359

[62] FDA. http://www.fda.gov/downloads/AboutFDA/CentersOffices/CDER/UCM203014.pdf&keyword=hiv-viral-load-management Accessed March 2016.03.31

[63] Daniels MJ, Hughes MD. Meta-analysis for the evaluation of potential surrogate markers. Stat Med 1997; 16: 1965–1982 9304767 10.1002/(sici)1097-0258(19970915)16:17<1965::aid-sim630>3.0.co;2-m

[64] Phillips AN, Eron J, Bartlett J, Kuritzkes DR, Johnson VA, Gilbert C, et al Correspondence between the effect of zidovudine plus lamivudine on plasma HIV level/CD4 lymphocyte count and the incidence of clinical disease in infected individuals. AIDS 1997; 11:169–175 9030363 10.1097/00002030-199702000-00006

[65] Ware NC, Pisarski EE, Tam M, Wyatt MA, Atukunda E, Musiimenta A, et al The Meanings in the messages: how SMS reminders and real-time adherence monitoring improve antiretroviral therapy adherence in rural Uganda. AIDS. 2016 5 15;30(8):1287–94. 10.1097/QAD.0000000000001035 26807967 PMC4853242

[66] Haberer JE, Musinguzi N, Tsai AC, Boum Y, Bwana BM, Muzoora C et al. Real-time adherence monitoring with follow-up improves adherence compared to electronic monitoring alone: quasi-experimental analysis. 11th International Conference on HIV Treatment and Prevention Adherence 2016 11th International Conference on HIV Treatment and Prevention Adherence, May 9–11, 2016, Fort Lauderdale. Abstract #56. http://www.iapac.org/AdherenceConference/Adherence_2016.html

